# Rethinking benefit sharing in collaborative human genetic research from an Afrocommunitarian perspective

**DOI:** 10.3389/fgene.2022.1014120

**Published:** 2022-10-12

**Authors:** Cornelius Ewuoso, Allan Sudoi, Dorcas Kamuya

**Affiliations:** ^1^ Steve Biko Centre for Bioethics, University of Witwatersrand, Johannesburg, South Africa; ^2^ KEMRI Wellcome Trust Research Programme, Kilifi, Kenya

**Keywords:** benefit sharing, afro-communitarianism, humanness, friendliness, partiality, human genetic research, Africa

## Abstract

This article draws on reflections about humanness, friendliness and partiality, in the writings of Afro-communitarians to develop principles for thinking critically about *why* benefit sharing, *what may count as benefits* within the context of human research in Africa and the *limits* of the obligation of benefit sharing. Suppose the thinking about humanness, friendliness, and partiality in Afro-communitarianism were the foundation of human genetic research in Africa, then, individuals who have contributed to research or borne its burden would benefit from its rewards. This is even more important if participants have pressing needs that researchers and/or research institutions can help ease. A failure to aid sample contributors and data providers in need when researchers and research institutions can—as well as an indifference to the serious needs of contributors—are failures to exhibit friendliness in the relevant ways. Finally, though providing benefits to contributors can be an important way of showing humanity to them, nonetheless, this obligation is not absolute and may be limited by the stronger obligation of shared experience—to advance science. Studies are still required to inquire how well these norms will work in practice and inform regulatory and legal frameworks.

## Introduction

Benefit sharing is a concept that frequently occurs in discussions about research involving both human and non-human samples and data (Schroeder, 2007a). It (benefit sharing) is mentioned in or supported by many different organizations, including the United Nations Education, Scientific, and Cultural Organization (UNESCO), The United States National Bioethics Advisory Commission (NBAC), World Health Organization (WHO), Human Genome Organization (HUGO) and World Medical Association (WMA). This idea has also been endorsed and discussed in research guidance documents such as the Declaration of Helsinki and the International Ethical Guidelines for Biomedical Research Involving Human Subjects (World Medical Association, 2001; Council for International Organizations of Medical Sciences (CIOMS), 2016). Within the human genetic research context, this concept has been taken to mean the equitable allocation of genetic research benefits (and burdens) amongst contributors and users of genetic materials. ^a^For this reason, benefit sharing is distinct from therapeutic misconception that occurs when a participant believes that participation in research will be of immediate *therapeutic benefit* to the participant (Henderson et al., 2007). The main goal of research is to contribute to generalizable knowledge, implying that research may have direct or indirect benefits to participants. Moreover, in benefit sharing, benefits are not limited to therapies or interventions. Doris Schroeder (Schroeder, 2007): [p. 208] describes benefit sharing as “the action of giving a portion of advantages/profits derived from the use of human genetic resources to the resource providers to achieve justice in exchange, with a particular emphasis on the clear provision of benefits to those who may lack reasonable access to resulting healthcare products and services without providing unethical inducements.” Benefit sharing raises different challenges in different contexts. Within the context of human genetic research *in Africa, particularly those conducted in international collaborative research*, the appeal of benefit sharing is that it can significantly reduce the risk of exploitation^b^ (Schroeder and Gefenas, 2012). This article does not claim that international collaborations are bad or that HICs are out to exploit African researchers and LMICs. There are other appeals of benefit sharing such as counter-balancing power differences that may exist between researchers or sponsors from HICs countries and contributors of genetic materials (who may be from LMICs), and the effects of commercialization.

Notwithstanding this apparent appeal, the practical implementation of benefit sharing is challenging. More so in human genetic research, where it raises important questions about the primary responsibilities of researchers and the nature and source of these responsibilities, as well as the limits, *if any*, of benefit sharing. Within the context of human genetic research in Africa, benefit sharing also raises questions about what should count as benefits and the responsibilities of international collaborators to African researchers and contributors.

This article draws on key concepts—humanness, friendliness and partiality—in the writings of Afro-communitarians to describe norms for thinking critically about the nature of obligations of benefit sharing and the limits of these obligations, specifically within the context of human genetic studies in Africa that are conducted in international collaborations. We do not however claim that these key concepts are unique or can only be found in African scholarship. Evidently, scholars in the Global North and East may have developed scholarship around these or similar concepts. Equally, an analysis could reveal that these concepts and how they have been understood in the Global North underlie guidelines like Nuremberg Code, the Declaration of Helsinki, the Belmont Report or the CIOMS guidelines. But we focus on how African scholars have understood these concepts. In other words, it is the African thinking about these concepts we draw on. As Thaddeus Metz (METZ, 2010), [p. 50] explains, “despite the lack of something utterly geographically distinctive, it is apt to call the [concept] African because the ideas that it expresses and that inform it are much more salient there than [elsewhere].” This methodological approach is important since it responds to the growing call for research in Africa to be informed by intuitions, modes of encountering the world and cultures dominant in the Global South (Branson, 2008; Campos-Mercade et al., 2021; Tuck and Yang, 2021). Also, we do not claim that humanness, friendliness and partiality are the only African concepts that we can draw on to address the objectives of this article. We focus on these since they occur frequently enough in the writings of African scholars to be considered as good candidates for describing norms informed by intuitions dominant on the African continent (Metz and Murove, 2009; Molefe, 2016; Ewuoso and Hall, 2019; Ewuoso et al., 2022).

In this article, we use participants and sample contributors interchangeably to refer to donors of samples collected and/or used in research. Additionally, we use African moral philosophy, African moral theory, Afro-communitarianism and African relationalism interchangeably to describe the philosophy informed by values and beliefs dominant in Sub-Saharan Africa. It is common to think of this philosophy as a communitarian philosophy, prescribing communal relationships understood as a combination of sharing a way of life and acting to enhance one another’s life qualities (Ewuoso and Hall, 2019). Communal relationships are equally core in prescribing ethical duties, developing full moral status and having dignity, hence the maxim “a person is a person through other persons” (Ewuoso and Hall, 2019). Consider the following remark by the former chair of the South African Truth and Reconciliation Commission, the late Archbishop Desmond Tutu (TUTU, 1999): [p. 35], “Social harmony is for us the *summum bonum*—the greatest good. Anything that subverts or undermines this sought-after good is to be avoided like the plague.” Though friendliness, humanness and partiality are not unique to the continent and may be found elsewhere, the moral intuitions that inform the thinking around these values in the writings of Afro-communitarians are more salient in sub-Saharan Africa and have not come to the continent from elsewhere. Similarly, though we allude to the *writings of African scholars* or *Afro-communitarians* in this article, this does not imply that all African scholars believe this to be true.

Equally, important to state at the outset is that benefit sharing within the context of international collaborative human genetic research raises important questions about the responsibilities—and the nature of these responsibilities—of researchers (who utilize samples) to their participants (who donate samples for research). It equally raises important questions concerning the responsibilities of researchers in high-income countries (HICs) to their collaborators in low/middle-income countries (LMICs) and the responsibilities of sponsors to researchers. Summarily, there are multiple stakeholders in human genetic research, thus implying that questions about distributing benefits and burdens will arise at several levels. Nonetheless, we focus primarily on what the thinking around humanness, friendliness and partiality in the writings of African scholars implies for the duty to share research benefits and the types of benefits to share with African participants and sample providers.

## Discussion

To realize the main goal of this article, we proceed to demonstrate for *the first time* ^c^ how African thinking about humanness, friendliness and partiality can inform norms for reflecting critically about *why* benefit sharing, *what may count as benefits* and the *limits* of the obligation of benefit sharing respectively (in human genetic research in Africa). Notice that the primary concern of the first section is to outline norms for thinking critically about benefits and benefit sharing. In this regard, this article is different from other studies like those published by Bege Dauda and others (Dauda and Dierickx, 2013; Dauda and Dierickx, 2017; Dauda and Joffe, 2018) that have mainly reflected on ethical issues around benefit sharing by drawing on dominant principles in the Global North. The article is equally different from other descriptive and explorative studies like those carried out by Nchangwi Munung and Jantina de Vries (Munung and de Vries, 2020), which describe opinions and views of participants regarding benefit sharing. This article is mostly normative, describing what the thinking about humanness, friendliness and partiality in African philosophy imply for what ought to be the case regarding benefit sharing. This approach is especially important since gaps exist regarding *types* of benefits and what may be beneficial in human genetic research in Africa. In the second section, we draw on the outlined norms to demonstrate their implications for benefit sharing, while addressing potential objections that 1) contend that research ought to be motivated by altruism and benefit sharing will attenuate contributor’s willingness to take part in research, 2) contend that benefit sharing will raise important challenges for informed consent, and the exact ways, 3) challenge the norms which we described, claiming that these norms have no relevance for the core issues (like undue inducement, under-compensation, *who* should actually benefit and implementation) regarding benefit sharing more broadly.

## Proposed norms for thinking about *benefit sharing*

### Humanness in afro-communitarianism and justifying benefit sharing

In African scholarship, humanness is sometimes differentiated from personhood. There are African scholars who believe that while humanness is gained through biological birth by humans, biological birth alone is not sufficient to gain personhood. Personhood is not biologically inherited. In addition to biological birth, individuals are required to act morally by prizing communal relationships to gain personhood. For example, in Masolo’s (Masolo, 2010) view, the notion of a person does not seek to distinguish a person from a non-person. It is an ideal towards which one strives rather than a status that one attains. Also, consider the statement by Menkiti (Menkiti and Wright, 1984): [p. 173]: “the African view reaches … for what might be described as a maximal definition of the person. As far as African societies are concerned, personhood is something at which individuals could fail, at which they could be competent or ineffective, better or worse.” The preceding statements contrast opinions expressed by leading African scholars such as Kwame Gyekye (Gyekye, 1992), who contend that personhood is not acquired. Accordingly, individuals are human persons first by virtue of biological birth before becoming or acquiring anything else. In Gyekye’s (Wiredu and Gyekye, 1992): [p. 108, note 22] words, “a human person is a person whatever his age or social status. Personhood may reach its full realization in community, but it is not acquired or yet to be achieved as one goes along in society.” Whilst Gyekye’s description of who a human is may share some similarities with views in the Global North, the reader should observe that the intuition that underlies Gyekye’s thinking is more dominant in the Global South than in the Global North. Particularly, personhood can only reach its full realization through sharing communal relationship (Ewuoso and Hall, 2019).

We draw attention to this debate, not to endorse a particular view, but to point out that not all African scholars think that humanness and personhood are always the same. This information has intrinsic value. Notwithstanding the debate about whether humans are already persons, most African scholars agree that humanness is a basic moral good, what individuals ought to be and how they ought to live. Individuals demonstrate that they are moral, and can increase, showcase *more* humanness, or fully express the same by acting in certain ways. Precisely, sharing a way of life and acting for the benefit of others. It is common to express this idea using the term *ubuntu* in southern Africa. As Mogobe Ramose remarks, to be a human being is to affirm one’s humanity by recognizing the humanity of others and, on that basis, establish humane relations with them. Ubuntu, understood as be-ing human (humanness); a human, respectful and polite attitude towards others constitutes the core meaning of this aphorism (a human is a human through other humans) (Ramose et al., 2002): [p. 231].

Part of establishing humane relations with others (and thus, showcase humanity) include fostering individuals’ capacity for communal relationships, honouring their values or means by which they have dignity, empathizing with others, responding to their basic needs—especially when one can, reciprocating the good done by others and cooperating with them to realize shared ends. Precisely, the ultimate goal of the biological human ought to be to become a genuine human being, i.e., to exhibit positive actions and virtues that humans can exhibit and in a way that not everyone may end up doing (Metz, 2010a): [p. 83]. A failure to exhibit positive actions towards others entails a failure to be human. Similarly, a failure to respect other humans is a failure to respect one’s humanity*.* Many Africans would say of those who fail to be humans that they are animals (Metz and Michalos, 2014).

One principle that emerges from the thinking about humanness in the writings of scholars of Afro-communitarianism is that one ought to establish respectful, humane relations with other humans since this is the basis of showcasing or becoming more human oneself. Within the context of this paper, one way of showcasing humanness to participants includes reciprocating research participation (Lefa, 2015). Other ways include compassion, respecting the dignity of others and treating people right. Concretely, suppose sample contributors have borne the burden of research, part of treating them right could reasonably include sharing research benefits and outcomes with them. This is congruent with Schroeder’s (Schroeder, 2007b): [p. 208] claim that benefit sharing is the action of giving part of the research advantages to contributors *to achieve justice.*

Similarly, if communities and nations have borne the burden of research in some way, part of treating them right/humanely could entail sharing research benefits and outcomes with them. Based on the Afro-communitarian obligation of showcasing humanness to one another, exploitation would be considered immoral. Whenever research is done collaboratively, the research question needs to respond to the needs of all collaborators and should not be skewed to answering research questions that are only important to the collaborator that has more power or resources. Summarily, benefit sharing is a matter of showcasing humanity between primary sample contributors and secondary users; or within the context of collaborative genomic research in Africa, between partners from HICs with their counterparts in LMICs. This obligation is even more important when sample contributors are in need (financial, health, and/or social needs) but lack resources to address those needs. An important aspect of showcasing humanness to others—is responsiveness to their needs. The indifference to the needs of others could be considered a failure to showcase humanity. On this view, humanness in itself, is an important justification for benefit sharing.

### Friendliness and understanding what could count as *benefits*

Like humanness, friendliness is also a core value in African philosophy. Nevertheless, we acknowledge that scholarships have been developed around friendliness in other regions. As an example, Aristotle has developed *corpus* of work on friendship. But we focus on the African thinking about this concept to respond to the call to shift research in Africa to Africa. For Tutu (1999): [p. 35], “We say a person is a person through other people. It is not I think therefore, I am. It says rather: I am human because I belong. I participate I share …… Harmony, friendliness, community are great goods.” The thinking is that friendliness is *good for its own sake* and requires a *combination* of identifying with others and exhibiting goodwill towards them. To identify with others roughly implies developing a sense of togetherness with others, whilst exhibiting goodwill roughly implies caring for their quality of life and acting in ways that are more likely to improve their wellbeing. Individuals become more or less of a human to the extent that they prize friendliness.

For scholars like Thaddeus Metz, the combination of identifying and exhibiting goodwill is what distinguishes *Afro-communitarianism* from solely teleological or consequentialist principles. As Metz remarks:

A moral theory that focuses exclusively on promoting good outcomes however one can (which is “teleological”) has notorious difficulty in accounting for an individual right to life, among other human rights. I therefore set it aside in favour of an ethical approach according to which certain ways of treating individuals are considered wrong at least to some degree “in themselves”, apart from the results. Honouring communal relationships would involve, roughly, being as friendly as one can and doing what one can to foster friendliness in others without one using a very unfriendly means. This kind of approach, which implies that certain ways of bringing about good outcomes are impermissible (and is “deontological”), most promises to ground human rights (Metz, 2011): [p. 540].

Part of promoting friendly relationships entails being friendly to those who have been friendly and exhibiting proportional unfriendliness towards those who have been unfriendly. Friendliness is also useful for thinking about why contributors may be owed an obligation of benefit sharing. Suppose sample contributors, participants and data providers have exhibited friendliness towards researchers by cooperating with them to contribute to generalizable knowledge. Then, researchers have an obligation to exhibit friendliness back to the participants. This is also treating participants right. Many participants in human genetic research in African countries may live in poverty or have unmet healthcare needs. Ensuring that research addresses the health needs, or the material conditions of participants may be one way of exhibiting friendliness.

Though *ubuntu* tends to encourage friendliness generally, this philosophy considers that the opposite of friendliness, that is, unfriendliness^d^ may be permissible when it is necessary to end proportional unfriendliness. In other words, there are different ways one can be unfriendly. But not all unfriendliness is necessarily impermissible. To be unfriendly is to exhibit ill-will and may include the use of coercion. There are also other more extreme forms of unfriendliness like deep hatred, violence, and generational enmity, which ought to be treated differently. Involuntary hospitalization and involuntary treatment are some forms of unfriendliness that may be justified if this is necessary, for example, to address mental illness (Ewuoso, 2018). Contrarily, they will be unjustified suppose there are no mental illness or any illness to address (Metz, 2011). For the purpose of this discussion, we focus on soft forms of unfriendliness like exploitation.

Suppose certain ways of promoting good outcomes are impermissible. In that case, one could not use unfriendliness to promote friendliness. This will not be honouring the value of friendliness. It also implies that one could not use very unfriendly means (or substantial unfriendliness) to end unfriendliness. Suppose one could disarm an aggressor and prevent unfriendliness to oneself by simply taking away the knife in the aggressors’ hand, then one is not justified to kill the aggressor. This will be using a very unfriendly means. Unfriendliness is permissible to the extent that it is necessary and sufficient to counteract a proportionate discordant behaviour. In this regard, unfriendliness towards those who have not been discordant will be immoral. Precisely, given that participants may not always comprehend research information, the African thinking about unfriendliness can usefully prevent exploitation in benefit sharing since exploitation is a form of unfriendliness towards those who have been friendly.

The preceding view of friendliness in Afro-communitarianism suggests that actions are right to the extent that they prize friendly relationships and not discord or enmity. There are other normative principles for thinking critically about benefit sharing that emerges from the description of friendliness in the writings of African scholars. Another principle that emerges is that when it comes to relating with participants and sample providers and/or their communities, the thinking that instructs one to value friendliness would normatively imply that researchers ought to think about and be responsive to the wellbeing of participants rather than be indifferent to their needs or act discordantly towards them.

Friendliness is also useful for thinking about *what could count as benefits* for Africans. Regarding the benefits to be shared with participants and sample providers, one model is the reasonable availability model, which limits benefits to those directly derived from the use of contributed materials. For instance, the Ethics Committee of the Human Genome Organization proposes (in its statement on benefit sharing) that for-profit entities could set aside between 1–3% of research profits for projects in host communities. The justification for the reasonable availability model is that given the risk of exploitation in human genetic research, ensuring that contributors benefit during research or from research outcomes (post research obligation) may be one way of reducing the risk of exploitation, increasing the social value of research or community’s bargaining power and ensuring that neglected diseases are given priority. This may be relevant for genomic research in Africa, as hesitation about sharing samples in African genomics is sometimes associated with concerns about exploitation by HICs (Munung et al., 2021).

A second model is the fair-benefit model, which says benefit sharing may not be limited to those advantages directly derived from research. Participants may also negotiate for other types of advantages that they prefer (Dauda and Joffe, 2018). This negotiation may be complex and could take place over time. These advantages or benefits could include monetary benefits, household supplies, treatment access, royalties on interventions/drugs, or other non-monetary benefits like technology transfer, job creation, provision of research findings/results and capacity building (Sudoi et al., 2021).

Based on the philosophy that asks us to honour friendliness, the relevant benefits would reasonably be those that can enable individuals to identify with others and exhibit goodwill in the relevant sense. In other words, suppose African scholars believe that individuals have dignity by virtue of their capacity for friendliness or communal relationship (Ewuoso and Hall, 2019), then what counts as *benefits* ought to promote the *values of beneficiaries* or conditions that make communal living/life—or the capacity for it—possible. The goal of benefit sharing should not merely be to make individuals well off or wealthy, “but also to make them better people” (Metz, 2020): [p. 62].

Concretely, some relevant goods that participants and/or their communities in Africa can benefit from include clinically actionable findings since these can enhance contributors’ quality of life and/or their capacity to relate well with others. Additionally, interventions from research ought to be accessible by contributors and/or their community at a subsidized cost or free. What could equally be shared are research outcomes that can repair damaged friendliness. Pandemics and epidemics are events that can disrupt social harmony. Suppose research contributes to ending a pandemic, then it should be shared with the community and/or individuals who contributed to this knowledge. Other benefits worth sharing with contributors in African human genetic research may include those that can help contributors develop new forms of friendly relations and/or enhance existing friendliness. In this regard, benefits could aim to address poor social infrastructures to enable more friendly interactions. Benefits may also entail building capacity in host communities by way of scholarship grants and research training for the host community. The point here is that benefits should not be limited to only clinical benefits or those that directly accrue from the research. Rather, benefits should be considered as anything that fosters friendliness. Since the contributor/community is in the best position to determine what can foster its view of friendliness, meaningful engagement with communities, in which possible relevant benefits are discussed ought to be carried out. Essentially, the thinking about *what could count as benefits* for Africans highlights the importance of community engagement in conceptualising research (priorities) and agenda.

### Partiality and limits of benefit sharing

Partiality can help us think critically about the limits of benefit sharing. One view of partiality describes it as a description of the quality of feelings that an individual has towards others. Such as in the statement, she is partial to him. The aim of this section is to describe how Afro-communitarians describe this term. It is common to describe Afro-communitarianism as a partial philosophy (Ewuoso and Hall, 2019). The reader should note that not all African scholars necessarily believe this to be true. Some scholars exist who defend a contrary position (Etieyibo, 2017; Gyekye, 2003). In the same vein, calling African moral philosophy a partial philosophy does not imply that a philosophy needs to be African to be a partial philosophy. Indeed, there are non-African moral theories that are partial. Some examples include subjectivism and ethical egoism. Finally, though we appeal to a partial theory to think critically about the limits of the obligations of benefit sharing, we do not mean to imply that such intuitions can only be supported by partial theories. Accordingly, impartial moral theories are not necessarily doomed by their impartiality. Non-partial theories may have intuitions that cohere with partial ones. For instance, utilitarianism, which is an example of a non-partial theory may be able to reach the same conclusions as a partial theory. The appeal to the formulation of African theory as a partial one is not that no other theory can help us understand the limit of the obligations of benefit sharing. This appeal is informed by the need to contribute an underexplored African perspective to the discourse on an ethical benefit sharing. An African moral theory, rather than western ones, can better contribute this perspective.

Notwithstanding, the belief in African philosophy that a partial moral theory can better account for the partial intuition that we ought to save our family members before strangers. Blood and close ties are often considered grounds for having an obligation to aid. In some formulations of African theory, the closer the tie, the greater the obligation. The relevant maxims here are “family first, and charity begins at home” and “blood is thicker than water.” The idea is that we ought to favour those with whom we have a longstanding, ongoing, actual and current relationship over those with whom we have no longstanding relationship (or we have no relationship at all). As one scholar remarks: “It is unethical to withhold or deny botho/*ubuntu* towards a member of the family, in the first place and the community at large” (Wareham, 2017): [p. 131]. Friends and family typically fall in the category of those with whom we have a longstanding and/or actual relationship. For instance, we share biological materials with our family, which tie us stronger to them than to strangers. We have likely cooperated with our friends for longer than we have with strangers. In principle, we have a stronger obligation towards them than to strangers. In fact, in this instance, behaving partially towards those who have cooperated with us for long is a way of honouring reciprocal relations. In favouring those with whom we have a longstanding relationship, we perpetuate reciprocal relationships and foster community good. As Christopher Wareham (2017): [p. 136] remarks, “partial relationships make people happy and allow one to feel special; they contribute to better cooperation towards the common good: the agent’s happiness is increased when they increase the happiness of someone they know.”

Someone may point out here that a partial moral theory will inevitably favour counterintuitive duties. As an example, such a theory will condone nepotism. This fact about African moral theory has already been acknowledged by African scholars like Mogobe Ramose (Ramose et al., 2003). A critic may point out here that partiality implies that when a health professional is faced with conditions that require distributing limited resources, he has an obligation to prefer friends and family rather than use need or severity of the condition to determine who gets the limited health resource. This form of nepotism dishonours the value of friendliness or the requirement to develop human relations with others. By preferring one’s existing relationships over future ones, we thereby fail to exhibit friendliness towards all. The critic may conclude that nepotism ought not to be endorsed since it conflicts with many people’s (Africans and non-Africans alike) considered judgement.

In response, one way to reply to the critic is to claim that it is not the case that researchers ought to be partial in principle but fulfilling their impartial obligations might entail behaving partially. This would be an indirect acknowledgement that Afro-communitarianism is not a partial theory, a position many African scholars tend to reject. A more reasonable response to the critic’s concern about nepotism would be to point out that whilst partiality is encouraged in Afro-communitarian philosophy, it is not all that matters. Precisely, the encouragement for individuals to value actual, existing relationships over future ones does not imply that strangers count for nothing. In other words, a strongly partial moral theory inevitably leads to nepotism. Strong partialism undermines the requirement of friendliness through developing a sense of togetherness and will likely promote friendly relations through unfriendly means. But African moral philosophy favours moderate partialism, and in this way, avoids nepotism. In other words, African philosophy is impartial in some way, enjoining one to value friendliness and showcase humanity to all. In this regard, it is not the case that we no longer have an obligation to help strangers. We owe an obligation to all humans, simply by virtue of their humanity. At another level, Afro-communitarianism is partial, deeming the obligation towards those with whom we have an actual or longstanding relationship to be more binding in principle than our obligations towards strangers (Wareham, 2017). The bond with family is better established and longstanding and failing to honour this relationship tends to be more disrespectful of friendly relations than the failure to be friendly towards strangers. Similarly, the obligation towards fellow citizens is more binding than the obligation towards foreigners. And the obligation towards humans is more binding than the obligations towards non-humans, such that we ought to favour humans over non-humans, especially in dilemmatic situations where we have to choose between respecting humans and respecting non-humans, but we cannot do both at the same time. So, family first and charity begins at home is not all that matters. The greater obligation of friendliness limits partiality in ways that prevent nepotism. Given the norm never to use unfriendly means to promote friendliness, a government official ought not to redirect resources to his family and close associates. Such a government official will inevitably fail to develop a sense of togetherness with the majority of the citizens. This will be using unfriendliness towards the public (who have an equal claim to public goods but are subordinated and coerced into paying taxes that only benefit the officials’ relatives) and other public officials who have avoided nepotism (that is, unfriendliness) in fulfilling their duties (Metz, 2010b).

The point here is that some forms of partiality threaten friendliness and should be avoided, whilst other forms of partiality do not threaten friendliness. Since not all forms of partiality necessarily threaten friendliness, a government may still act partially say towards those wronged in the past by the State. Government officials have an obligation to act this way even when this will cost the public. The thinking that applies here is that we have an obligation to end or address unfriendliness before seeking new ones. Based on this account, victims of the State’s past unfriendliness may have a greater claim to the preferential treatment than others.

The normative principle that arises from the African view on partiality is that one has differentiated obligations towards those with whom we have certain types of relationships. The obligation towards sample contributors diminishes in intensity the less longstanding the relationship. By establishing a shared experience with the participant, a researcher has created a morally significant relationship with the participants (and *vice versa*) that requires the parties in the relationship to care for each other’s quality of life, which as Thaddeus Metz (2010): [p. 56] points out “can go beyond those listed in a contract [or consent forms].” The deeper and/or longer this relationship, the stronger the obligation towards the participants (than to non-participants). Yet, research creates a relationship not only between a researcher and a sample contributor but also between the researcher and the sponsors and between a researcher and her institution. If the researcher is collaborating with other researchers and institutions in other regions, a relationship also exists between the researcher and the other researchers/institutions. Where there is some relationship, there is some obligations. Researchers ought to be biased towards those with whom they have a relationship and act partially to promote their good.

The nature of the shared experience also limits this obligation. If a researcher and a contributor cooperate to advance science, this implies that the shared experience (that is, to contribute generalizable knowledge) has priority over advancing the contributor’s healthcare. Suppose the human gene contains information not only about the individual who contributed the sample but also those biologically related to the individual, there is, therefore, an obligation towards those relatives. In fact, someone may point out that there is a sense in which we share genetic material with all humans, thus implying that there is an obligation of benefit sharing to all humans (Pullman and Latus, 2003). The principle of partiality implies that a researcher’s obligation to sample contributors’ relatives and to all others is limited by the greater obligation to advance science and the more important obligation towards the contributors. The thinking here is that those who have borne the greater burden of *actually* contributing to research ought to receive preferential treatment over others. Suppose fulfilling the obligation to relatives will undermine the researcher’s capacity to advance science or fulfil the duties to participants. In this case, the obligation to relatives does not have priority.

It is our preposition therefore, that partiality, considered alongside friendliness, is an important guide on who is owed benefits from genomic research in Africa and in thinking about the limits of those obligations. We demonstrate this in the next section where we address specific objections against our position.

## Addressing possible objections and implications of the proposed norms

This section addresses some potential objections to the norms that we described in previous sections. Our responses draw primarily on Afro-communitarianism for the same reason we focus on thinking about humanity, friendliness and partiality in Afro-communitarianism, that is, to respond to the call to decolonize research in Africa. By drawing mostly on Afro-communitarian references, we do not imply that international references are irrelevant. Instead, we draw on Afro-communitarian sources given that these sources (more than international ones) can usefully respond to this call.

### Altruism and human genetic research

A critic may point out that participation in research ought to be motivated by altruism. Most participants recognize the importance of volunteering to help others or contribute to medical progress (Al-Ebbini et al., 2021). Studies show that some participants take part in research not necessarily because they aim for some benefits for themselves, but the wellbeing of their group (Berg, 2001). Such altruism is of real value to human genetic research that is often expensive. Structures for benefit sharing may be so coarse that it makes research prohibitively costly and thus, discourage sponsors from funding research thereby undermining global efforts to develop interventions that limit disease burden (Schroeder and Gefenas, 2012). In other words, most genetic research would not occur without the altruism of research participants. Altruistic attitudes are ways of exhibiting solidarity with others and, thus, of great value to society. If research leads to interventions that address ailment, this potentially benefits others. Contrarily, the emphasis on sharing research benefits could attenuate contributors’ willingness to participate in research, increase the cost of research and, by extension, the capacity to conduct research at all or undermine the scientific value of research since participants may refuse to disclose information that may jeopardize their participation in research (Lairumbi et al., 2012).

In response, notice that it is not the case that participants *are always* motivated to participate in research for altruistic reasons. In fact, studies show that participants are more motivated to participate in research when they think some benefits will accrue to them (Mein et al., 2012; Kamuya et al., 2014). In one study, participants mentioned that the primary reason for participating in the study “was the immediate access to high quality care” (Kamuya et al., 2014): [p.9]. Many human genetic research will eventually lead to profitable/patentable products/interventions (Pullman and Latus, 2003). Suppose research ought to be informed by the values of the people. In this case, it would be unethical not to reciprocate those who contributed to the research since reciprocating is a vital way of showcasing humanness. The African thinking about reciprocal relations is that they are relationships of mutual aid that honours the obligation of showcasing humanness. The relevant saying is, “the right arm washes the left arm and the left arm washes the right arm.” Equally consider the following remark by a key contributor to African socialism, Julius Nyerere (Nyerere, 1968), “In our traditional African society, we were individuals within a community. We took care of the community, and the community took care of us. We neither nor wished to exploit our fellow men.” Assuming a commercial profit was made from their contributions, participants in one study mentioned that they feel they ought to share in the profit (Moodley et al., 2014). So far, our response may appear to suggest that we favour post-trial benefit sharing. But assuming a researcher conducting research on new vaccines for HIV in a community that is equally burdened by malaria has access to proven interventions for malaria; the obligation to foster friendliness and showcase humanity to others would imply that this researcher ought to benefit the host community with those proven interventions. Hence, participants and their communities can be benefited during the study or even before the study commences. We acknowledge that benefiting participants will likely increase research cost. But the thinking about the right way to cooperate or exhibit friendliness implies that cost ought to be borne by all stakeholders in research (sponsors, research institutions, *etc.*) and factored into new interventions that are developed as a result of the research. Precisely, all potential beneficiaries of the knowledge that is generated from research ought to participate in one way in bearing its cost.

### Informed consent and African intuitions

Another objection may be that benefit sharing will raise significant challenges for informed consent. Best practice requires that participants and sample contributors be informed of the relevant aspects of research (Mello and Wolf, 2010). This may reasonably include any new secondary use of their samples. Suppose future uses of samples—for example, deposited in a biobank—are unknown at the time of consent, then it would be difficult to provide comprehensive material information about possible uses of contributed data (including likely risks/benefits associated with these uses) to participants. It may be difficult for researchers to know ahead of time *what types of secondary studies* will be carried out on samples/data. Who will have access to the data or the samples, for what purpose and indeed the kinds of benefits to expect? Issues around informed consent are further complicated when data from contributed samples are used for commercial purposes. Sometimes commercialization may not be anticipated (by researchers or research team) at the time of consent or sample taking. Some participants have indicated that they would be unhappy if their samples were re/used to generate profit in the future or exported to other regions (Al-Ebbini et al., 2021). The potential for commercialization raises questions about sharing profits with original owners of data. What rights would sample contributors have to share in the profit made by commercial entities using their samples? Similarly, how do we manage benefit sharing issues if the potential for commercialization was not mentioned during the informed consent process or where participants have not provided explicit consent for commercialization?

In an ideal research context, participants would be informed and asked to reconsent to every new use of their samples or data. Previously unanticipated potential for profits will equally be discussed with them as they arise. But there may be practical challenges that would make recontacting participants impossible. These challenges also apply to tiered consent. For instance, sample contributors may have expressed their wish not to be recontacted or may have died. One way of addressing this ethical challenge is for research participants to provide consent for future *unknown* use of samples. A profit-sharing formula will equally be discussed with them in anticipation of a likely commercialization of their data. Many guidance documents like the European Union’s General Data Protection Regulations and South African’s Protection of Personal Information Act (POPI Act) allow such broad consent. The thinking here is that it is unethical not to carry out previously unanticipated research that will benefit society, or that participants themselves can eventually benefit from (Moodley et al., 2014). POPI Act allows for future uses of samples so far as such uses are *compatible* with the initial reason for collecting the sample (Staunton and De Stadler, 2019). This is important for genomic studies conducted in Africa, given the significant underrepresentation^e^ of African genomes in the genomic data pool, and thus implying limited (if any) benefits to Africans (Moodley and Kleinsmidt, 2021; Munung et al., 2021).

But it is not clear how “compatible” purpose may be interpreted: narrowly or broadly. Is this limited to research on the disease for which sample was collected such that research on other diseases will be incompatible or research on any disease such that using the specimen to generate profit will be incompatible? What African thinking about friendliness suggests about compatible purpose may be that such use should honour the values of contributors or foster the capacity for friendliness. Note that contributed samples may be tagged with donors’ preferences, including the agreed profit-sharing formula in the event that their data generate profits. In this case, research that aims to eliminate the disease burden that affects contributors could reasonably fall within the purview of compatible purpose. This would likely enhance their quality of life and, thus, the capacity to relate well with others. However, broad consent raises other challenges. Importantly, we acknowledge that it is hardly possible that consent can be informed if it is for future unknown use. Moreover, if a profit-sharing formula is drawn-up before one has full knowledge of what the profit might be, this could mean that a participant who could have received more may get less (or *vice versa*). If they get less, this will not be treating the participants right. Moreover, what does broad consent mean within the context of genomic research in Africa, where samples are often shipped off to foreign institutions where it may not be possible to control how samples are then used or benefits accrued shared? Some national Acts like the South African National Health Act require that where participants’ preferences regarding secondary or future uses of contributed samples are unknown, the researcher ought to approach the Ethics Committee to request approval for new uses of samples (Moodley and Kleinsmidt, 2021). In one study, about 95% of participants mentioned that they are happy not to be recontacted for new and secondary use of their samples insofar as such new use was approved by a review committee (Wendler et al., 2005). Where participants’ preferences are unknown, the African view of partiality suggests that the nature of the shared experience ought to guide future use of samples. Suppose researchers and participants collaborate to advance science by contributing towards generalizable knowledge, then contributed samples or data from samples ought not to be used for commercial purposes, at least without contributors’ consent. The nature of the shared experience ought to be made available to secondary users of samples. Suppose a researcher does not know if data from samples would be used for commercial purposes in the future at the time of consent. In this case, a researcher should consider discussing the possibilities of commercialization, royalties, and unknown future use of data with participants at the time of consent, especially given that most human genetic research will likely lead to commercial products (Pullman and Latus, 2003). This will be a way of treating participants right or showcasing humanness to them. Moreover, it seems intuitive that a reasonable person would consider such material information relevant. This way too, participants and sample contributors can indicate their preferences. It is also a way of honouring individuals’ capacity for friendliness. Specifically, developing a sense of togetherness with others and cooperating with them requires that one is clear or transparent to the extent possible about the terms of the relationship. Participants’ capacity to be friendly with others or to genuinely cooperate is undermined if they do not have sufficient information about the terms of the friendliness.

## Challenging the African-inspired obligation of benefit sharing

Whilst benefit sharing can occur at the outset of the study, implying that participants may be benefited at the beginning of the research (Berg, 2001), there are ethical problems with mandating benefit sharing at the beginning of the study. Apart from uncertainties about what benefits can be shared at this point, a critic may point out that this will lead to undue inducement. Another critic may also add—as we have observed in the previous section—that if benefit sharing takes place at the beginning of the study, this may imply that participants who could have benefited more from very successful research would receive less. Post-study benefit sharing obligations are also not without certain challenges. How do you share benefits if the original contributors of genetic samples are unknown? Collected samples are often anonymized to reduce the risk of loss of confidentiality. In some cases, as is the case with many population genomic studies and with samples deposited in biobanks, a small number of samples may have been collected from various regions and over a long time. In these instances, how do we quantify contributions that deserve benefits, and in what ways? Who should receive benefits if participants are unknown or samples have been combined from several groups and regions? What importance does one sample have over others? In this case, discussing benefits with participants at the outset of the study may be problematic.

Furthermore, do samples have any inherent value or is the value created by the researcher? If researchers manipulate samples to develop interventions, should expert labour outweigh participants’ contribution or what is the limit of the obligation to share research benefits? It could also take several months or years after many attempts for a successful/new intervention to be developed from collected samples, meaning that benefits may not be known at the time of collection. The failure rates in intervention developments are also very high, and the probability that sample contributors will contribute to research that actually produces a profitable intervention is slim (Schroeder and Gefenas, 2012). In some cases, a new intervention may not result. There are also practical issues about implementation (Lairumbi et al., 2012). Who will ensure that benefits are actually shared with the participants and or community? The ethics committees in most African countries often lack the resources—including the infrastructure and training—to give adequate attention to benefit-sharing implementation post research period or the protection of human participants (Schroeder and Gefenas, 2012). Given the time it takes to create interventions, it may also be difficult for IRBs and the government to fulfil their oversight duty. Moreover, researchers may have left research sites or lost contact with participants, the host community, or the ethics committee when benefit sharing becomes relevant. Regulatory infrastructure equally need to be standardized to guide the implementation of sharing agreements and prevent individuals from implementing benefit sharing as they consider (Sudoi et al., 2021). Given these potential issues, a critic may challenge the relevance of the norms which we described, contending that these norms do not address the core issues around benefit sharing in human genetic research.

This potential objection raises questions about whether 1) benefit sharing may be ethical given the potential for under-compensation and/or undue inducement, 2) *who* ought to actually benefit if original contributors are unknown or if researchers are essentially responsible for manipulating samples to create value, and 3) *who should implement* or *how* does one realize benefit sharing? Questions about undue inducement and under-compensation are important. Undue inducement entails A exploiting the vulnerabilities like poverty of B to coerce B into agreeing to something which B would not have agreed to had B not been poor. One suggestion for addressing undue inducement and under-compensation is that benefits ought to accrue to the communities rather than to participants (Kamuya et al., 2014). Yet it is equally true that if participants in poor settings are denied benefits (say financial benefits) because their poverty makes them vulnerable, they would have suffered two-fold harm: poverty and being denied financial benefits. In other words, vulnerable persons would have contributed to research to which they cannot benefit from mainly because of their vulnerability. We acknowledge that questions about inducement or how much benefit is enough, require to be researched further. We propose to do this in a future study. However, our intuition about undue inducement and under-compensation is that these two have a lot more to do with 1) *power asymmetry* between parties (implying that one party has less power that renders him vulnerable to agree to unfair/inadequate benefits) and, 2) *what type of* benefits contributors receive. These can be addressed through an emphasis on friendliness. Notice that the problem here (especially about undue inducement) is not that *there is inducement at all* but that there is an *excessive offer* that distorts decision making, thus, coercing individuals into participating in research against their informed judgement (Molyneux et al., 2012). Suppose scholars of Afro-communitarianism believe that individuals have dignity by virtue of their capacity for friendliness. Then, it seems intuitive based on this thinking that contributors can hardly be under-compensated or unduly influenced if benefits aim to enhance their capacity for friendliness in the relevant ways, repair damaged friendliness, honour their values or means by which they have dignity. Collaborative partnership with the community and contributors in all aspects of the research will play a vital role in ensuring that benefits foster friendliness and that they (contributors and communities) are involved in key decisions about benefit sharing.

Regarding (2), what African thinking about partiality suggests is that if researchers have lost contact with the original contributors of samples, the benefits should accrue to their relatives, beginning with the closest relatives. Suppose the original contributors are unknown. In this case, benefits should accrue to their community or country. Notice that samples may be coded, rather than anonymized, to facilitate benefit sharing with original contributors.

The concern about whether the researcher’s manipulations of the sample are worth more than the sample itself, or whether a particular sample contributed to the development of the eventual intervention, misses the point of our argument, which is that those who have contributed to human genetic research ought to equally benefit. We acknowledge that both the contributed samples and the researcher’s manipulation are important. It seems intuitive that there would be nothing to manipulate if the sample does not exist. Scientists most often acknowledge that the key to, say, developing a new intervention for tuberculosis (TB) is the contributed sample (Berg, 2001). Suppose individuals who have this condition are unwilling to participate in research, then the capacity to improve the medical situation—through the development of (new) interventions—of those with TB is significantly undermined. This is true even with the fact that humans essentially share the same genome, specifically about 90% of their genome (HUGO, 2000). By requiring companies to set aside between 1–3% of their profits for charity, it seems the Human Genome Organization is acknowledging the primary importance of contributed samples. Similarly, the samples would not be manipulated if the manipulator is absent. Both seem to us important, and questions about which one is more important miss our primary point about the *obligation and responsibility*—*if any*—*of sharing research benefits with participants and sample contributors and why these obligations exist.*

Regarding (3), notice that we have mostly stated that researchers owe contributors an obligation of benefit sharing in this article. However, we do not think that only researchers have this obligation towards contributors or ought to oversee its implementation. We acknowledge that each nation and community ought to develop its own guidelines regarding *who* should ensure that contributors benefit by considering its context and available resources, including existing bodies that can oversee the implementation of benefit sharing. Nonetheless, the Declaration of Helsinki suggests researchers, sponsors and host communities/governments as those to comply with the obligations of benefit sharing (Mastroleo, 2016). The Brazilian National Health Council requires that access to the advantages that accrue from research must be guaranteed by the sponsor and/or the researcher’s institution (Schroeder and Gefenas, 2012). One may also add to this list the institutional review board that granted the ethics approval for the study, and governments of sponsors’ and the researcher’s country for publicly funded research. Pressure from communities and societies have also been shown to be effective in ensuring that researchers and institutions behave ethically towards contributors, implying that social pressure can be an effective means of ensuring that benefit sharing becomes a reality (Hurst, 2017). Essentially, the thinking here—which is informed by friendliness—is the African saying, “it takes a village to raise a child.”

## Conclusion

Key concepts in the writings of scholars in African philosophy have important relevance for benefit sharing. In this paper, we have demonstrated how ideas about showcasing humanness, exhibiting friendliness and partiality can usefully ground norms for thinking critically about *why* benefit sharing, *what could count as benefits* and the *limits* of the obligation of benefit sharing, within the context of collaborative human genetic research in Africa. First, the obligation to showcase humanity to one another would imply that if individuals have contributed to the research, borne its burden, they ought to equally benefit from its rewards. Second, this obligation is all the more important if participants have pressing needs that researchers and/or research institutions can help to ease. A failure to aid participants in need when researchers and research institutions can—as well as an indifference to the serious needs of participants—are failures to exhibit friendliness. Finally, the obligation to aid is not absolute and may be limited by the more important obligation to advance science. Studies are still required to inquire how well these norms will work in practice and be enshrined in legal and regulatory frameworks, especially if these norms are to be taken seriously.

## Data Availability

The original contributions presented in the study are included in the article/Supplementary Material, further inquiries can be directed to the corresponding author.

## Footnotes

a Given the nature of genetic materials, there may be other stakeholders in benefit sharing that can include family members of contributors, their communities, regulatory bodies, research institutions, sponsors, biobanks, the ethics committee, physicians who collect biosamples and countries sponsoring or hosting genetic research.

b Exploitation: Defined in this context as taking unfair advantage of someone for one’s own benefit.

c To the best of our knowledge, we are not aware of any similar article that has applied the same concepts that we draw on to inform norms for thinking about benefit sharing within the context human genetic research in Africa.

d Unfriendliness includes failure to idenfity with others or exhibit goodwill. Exploitation, coercion, cruelty, promise-breaking, stealing, murder are some forms of unfriendliness.

e Evidently, there are reasons for this underrepresentation. But this is outside the scope of this paper.
